# Relevance of Autophagy in Parenchymal and Non-Parenchymal Liver Cells for Health and Disease

**DOI:** 10.3390/cells8010016

**Published:** 2019-01-01

**Authors:** Ralf Weiskirchen, Frank Tacke

**Affiliations:** 1 Institute of Molecular Pathobiochemistry, Experimental Gene Therapy and Clinical Chemistry, University Hospital RWTH Aachen, D-52074 Aachen, Germany; 2 Department of Medicine III, University Hospital RWTH Aachen, D-52074 Aachen, Germany

**Keywords:** hepatocytes, hepatic stellate cells, sinusoidal endothelial cells, macrophages, fibrosis, cirrhosis, hepatocellular carcinoma, biomarkers

## Abstract

Autophagy is a highly conserved intracellular process for the ordered degradation and recycling of cellular components in lysosomes. In the liver, parenchymal cells (i.e., mainly hepatocytes) utilize autophagy to provide amino acids, glucose, and free fatty acids as sources of energy and biosynthesis functions, but also for recycling and controlling organelles such as mitochondria. Non-parenchymal cells of the liver, including endothelial cells, macrophages (Kupffer cells), and hepatic stellate cells (HSC), also employ autophagy, either for maintaining cellular homeostasis (macrophages, endothelium) or for providing energy for their activation (stellate cells). In hepatocytes, autophagy contributes to essential homeostatic functions (e.g., gluconeogenesis, glycogenolysis, fatty acid oxidation), but is also implicated in diseases. For instance, storage disorders (alpha 1 antitrypsin deficiency, Wilson’s disease), metabolic (non-alcoholic steatohepatitis, NASH), and toxic (alcohol) liver diseases may benefit from augmenting autophagy in hepatocytes. In hepatic fibrosis, autophagy has been implicated in the fibrogenic activation of HSC to collagen-producing myofibroblasts. In hepatocellular carcinoma (HCC), autophagy may contribute to tumor surveillance as well as invasiveness, indicating a dual and stage-dependent function in cancer. As many drugs directly or indirectly modulate autophagy, it is intriguing to investigate autophagy-targeting, possibly even cell type-directed strategies for the treatment of hereditary liver diseases, NASH, fibrosis, and HCC.

## 1. Introduction

**Figure 1:**
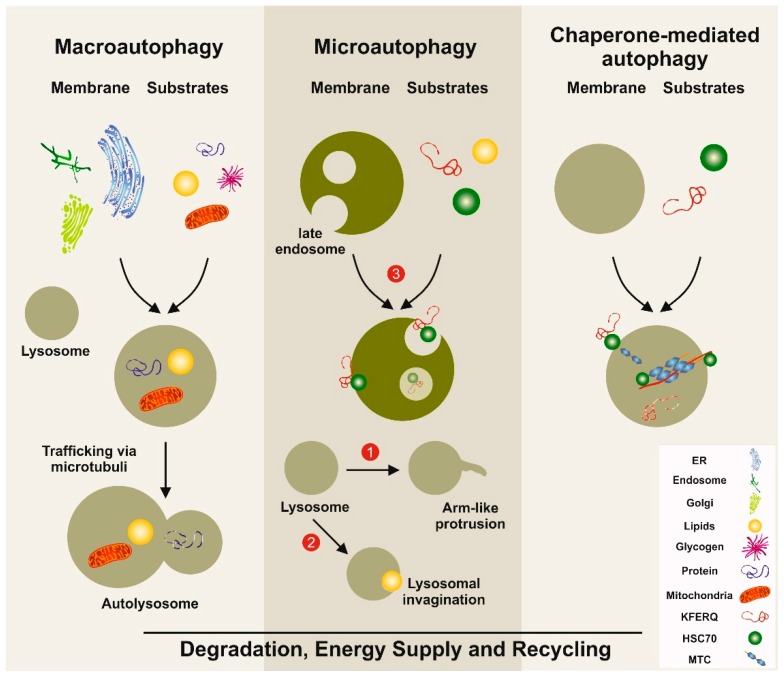
Simplified models of autophagy pathways in the liver. Macroautophagy involves the formation of a double-membrane vesicle, in which the substrates to be degraded are included. This vesicle called the autophagosome is then fused with the lysosome, allowing the degradation of the products. Three distinct types of microautophagy exist. In one type, the lysosome forms arm-like protrusions capable of engulfing substances. In a second branch, the lysosome can form invaginations, in which substrates (e.g., lipids) can be wrapped. The most important pathway in microautophagy involves the late endosome. In this compartment, substrates such as proteins carrying the pentapeptide lysine-phenylalanine-glutamic acid-arginine-glutamine (KFERQ)-like motifs are internalized and degraded. In chaperone-mediated autophagy, substrates with a KFERQ-like motif are first recognized by the cytosolic chaperone. Subsequently, this complex is recognized by chaperone-mediated autophagy associated receptors located at the lysosomal compartment. After internalization, the incorporated substances are degraded. The three autophagy pathways serve as a dynamic recycling system that produces new building blocks and provides energy necessary to guarantee cellular homeostasis. ER: endoplasmic reticulum; HSC70: heat-shock 70-Kd protein; MTC: multimeric translocation complex.

The term autophagy summarizes the processes involved in the orderly degradation and recycling of worn, abnormal, or malfunctional cellular components. It is commonly accepted today that the term “autophagy” was first introduced in 1963 by the Belgian cytologist and biochemistry Christian René de Duve, who also coined the terms “endocytosis” and “phagocytosis” to designate pathways bringing substrates for digestion in lysosomes [1]. However, the terms autophagy/autophagy/autophagia were in fact already used a century earlier and published in 1859 in a French journal [2]. The importance of autophagy was prominently acknowledged in 2016, when Yoshinori Ohsumi was awarded the Nobel Prize for Physiology or Medicine for his discoveries of mechanisms for autophagy. Autophagy is nowadays considered as a dynamic recycling system, which is essential for cellular renovation and homeostasis [3]. As such, the resultant degradation products can be used for new protein synthesis, energy production, and gluconeogenesis. There are three classes of autophagy, namely macroautophagy, microautophagy, and chaperone-mediated autophagy, requiring different sets of autophagy-related genes and cellular compounds [3] (Figure 1). Macroautophagy is the most prevalent form of autophagy. It is dependent on the “autophagosome”, a spherical vesicle appearing randomly throughout the cytoplasm with the capacity to traffic along microtubules towards the microtubule-organizing center, where lysosomes are concentrated [4]. These ring-shaped structures are majorly formed by the “AuTophaGy” (ATGs) genes that are evolutionarily conserved from yeast to higher eukaryotes. This cellular compartment has the capacity to sequester small portions of cytoplasm enriched in soluble materials and organelles and to fuse with lysosomes forming the autolysosome, in which the material is finally degraded. On the contrary, microautophagy is a more diverse type of autophagy, in which cytoplasmic compounds or spontaneous formed vesicles are directly engulfed by lysosomes. Recent studies demonstrate that this pathway is of particular relevance for cells under amino acid starvation [5]. Based on the finding that vascular membranes and endosomes can also incorporate or capture peroxisomes or lysosome-derived organelles, it was proposed that this autophagy branch should be classified in three distinct subtypes of microautophagy [6]. Chaperone-mediated autophagy is more selective and not associated with membrane reorganization [3]. Instead, chaperone and co-chaperone proteins recognize cytosolic proteins that carry specific peptide recognition sites and are then targeted to receptors on lysosomes, which subsequently internalize these proteins for degradation (Figure 1). This pathway majorly contributes to the maintenance of cellular homeostasis by facilitating degrading of proteins and recycling of amino acids. However, transgenic mouse models have shown that this pathway participates in the regulation of glucose and lipid metabolism, DNA repair, cellular reprogramming, and cellular response to stress [7].

With regards to the liver, there is strong evidence that the process of macroautophagy in particular is the most important for maintaining hepatic homeostasis and suppressing spontaneous tumorigenesis. The systemic mosaic deletion of *Atg5* in mice resulted in multiple benign tumors that developed only in the liver but not in other tissues [8]. On the other side, host-specific deletion of *Atg7* impaired the growth of multiple allografted tumors in mice, most likely by inducing release of arginosuccinate synthase 1 from the liver and degradation of circulating arginine, which is essential for tumor growth [9]. These inverse findings demonstrate that autophagy plays a dual role in cancer cells with potential to both inhibit and promote tumor progression and promotion.

In the present review, we will highlight some principal and cell-type specific functions of autophagy in the liver, its role in hepatic homeostasis, and its impact on the pathogenesis of liver diseases. In addition, we will discuss how the present knowledge in autophagy research might influence future directions in therapy of liver diseases.

## 2. Principal Functions and Molecular Mechanisms of Autophagy

Autophagy is an important conserved recycling process necessary to maintain energy balance in the cells. In the liver, the activity of this cellular autophagy activity is enhanced or reduced in response to environmental changes and cellular needs [10]. It is not only essential for replenishing the free pool of amino acids through protein breakdown, but it also contributes to mobilization and hydrolysis of lipid stores and glycogen, thereby significantly contributing to the cellular energetics and energetic flux through different metabolic pathways [10]. The occurrence of three different types of autophagy provides a high functional variety of possible breakdown and recycling processes, which are particularly relevant for the liver, which represents the central organ in the control of organismal energy balance (Figure 1). Consequently, alteration in proper autophagy function can result in severe metabolic disorders such as obesity, fatty liver, diabetes, and other metabolic age-related disorders [11,12]. Recent findings further suggest autophagy as a critical mechanism in regulating the “liver clock” and circadian glucose metabolism by timely degrading core circadian repressor clock proteins such as crytochrome 1 (CRY1), resulting in gluconeogenesis and increased blood glucose levels [13]. Interestingly, high-fat feeding decreased CRY1 protein expression in an autophagy-dependent manner, while restoring hepatic CRY1 reversed obesity-associated hyperglycemia, suggesting that this regulatory network is a potential attractive target for therapy of obesity-associated hyperglycemia [13].

There is also first evidence that autophagy in liver aggravates the oxidative stress response during acute liver injury. In particular, autophagy maintains liver endothelial cell homeostasis and protects against cellular dysfunction, intrahepatic nitric oxide accumulation, and a liver microenvironment that promotes fibrosis [14]. Similarly, the blockade of autophagy by the autophagy inhibitor LY294002 or small interfering RNAs (siRNAs) targeting *Atg5* attenuated drug-induced anti-inflammatory effects in hepatic stellate cells and on liver fibrosis [15].

Mechanistically, there is experimental evidence showing the PI3K/Akt/mTOR pathway to be critically involved in the activation of autophagy, thereby preventing cell death, promoting anticancer effects of therapeutic drugs, and reducing tumor growth [16]. On the contrary, in hepatocellular carcinoma (HCC) cells, the induction of the PI3K/Akt/mTOR pathway by α-fetoprotein (AFP) resulted in reduced cell autophagy and more malignant behavior [17]. These opposite findings demonstrate that the same autophagy-associated pathway are highly dynamic and can have pro-tumor or anti-tumor effects. Hence, the role of autophagy in HCC development is dependent on the context of liver cells, the hepatic microenvironment, stage of tumor development, or many other unrecognized factors. It is most likely that autophagy plays an anti-tumor role in normal liver cells by maintaining cell homeostasis, while it promotes the survival of HCC cells within the tumor microenvironment once the tumor is formed [18].

## 3. Autophagy in Homeostasis of the Liver—Implications for Hereditary Liver Diseases

The importance of autophagy for the maintenance of liver homeostasis is best exemplified in conditions, in which large quantities of misfolded proteins are formed that lead to an overburden of the proteolytic pathway involved in autophagy. Prototypically, patients suffering from classical α1-antitrypsin (α1AT) deficiency synthesize large quantities of mutant α1AT Z (ATZ) protein in which a point mutation results in a substitution of lysine for glutamate at residue 342 [19]. While the normal α1AT protein (M protein) is rapidly secreted into the blood, the missense mutation results in a polymerized mutant α1AT protein (Z protein) that is retained in the endoplasmic reticulum of hepatocytes rather than secreted in the body fluids where its physiological function is to inhibit neutrophil proteases [19,20]. Hepatocytes deal with the burden of insoluble aggregates by activating endoplasmic reticulum-associated proteasomal degradation pathways and by macroautophagy [21]. However, in most homozygous individuals these countermeasures are insufficient to overcome the overload with insoluble proteins, provoking cell death and chronic liver damage. The clinical manifestation of liver disease associated with α1AT deficiency is highly variable, and there is currently no specific treatment of α1AT-related liver disease [22]. Enhancing cellular degradation pathways, particularly autophagy, for mutant ATZ proteins may therefore represent a realistic option in the near future [23]. Independent experimental studies have shown that the induction of autophagic degradation of mutant polymerized Z protein by hepatic gene transfer of master autophagy regulators or by autophagy-enhancing drugs such as carbamazepine, rapamycin, or 24-norursodeoxycholic acid (norUDCA) can significantly reduce liver injury [21,24,25,26]. These approaches, along other targets (e.g., blocking mutant ATZ production by siRNA), are currently under clinical evaluation,

Another inherited disorder reflecting the importance of autophagy in liver homeostasis is Wilson’s disease, also known as hepatolenticular degeneration or “copper storage disease”. It represents a rare autosomal recessive disorder caused by mutation in the ATPase copper transporting protein ATP7B, preventing the body from removing excess copper and leading to accumulation of this trace metal in liver and brain [27]. Recently, it was shown that ATP7B-deficient cells showed significant increased expression of autophagy-associated genes when compared to control cells. Furthermore, hepatocytes derived from patients suffering from Wilson’s disease, as well as hepatocytes derived from *Atp7b* null mice and rats, contained elevated quantities of autophagosomes [28]. Interestingly, the pharmacological inhibition of ATG7 and ATG13 accelerated cell death in the hepatoma cell line HepG2 when depleted for ATP7B expression, suggesting that autophagy protects against metal toxicity and copper-induced cell death in the setting of Wilson’s disease [28].

Alcohol abuse is a third condition in which the importance of autophagy for liver homeostasis is well documented. Alcoholic liver disease (ALD) is a global healthcare problem associated with fatty liver, alcoholic hepatitis, fibrosis, and cirrhosis. During chronic ethanol consumption, the rates of autophagy are retarded in the liver, because ethanol is thought to cause faulty lysosome biogenesis and slower breakdown of lipid droplets [29]. A recent experimental study found that liver tissue from mice fed with ethanol displayed lower expression levels of total and nuclear transcription factor EB (TFEB) compared with control mice, alongside decreased parenchymal lysosome biogenesis and autophagy [30]. When the hepatic expression of the transcription factor TFEB was increased by administration of torin-1, representing an effective inducer of autophagy, or by administration of an adenoviral vector expressing TFEB, mice showed decreased steatosis and liver injury induced by ethanol, while the knock down of TFEB using an adenovirus small hairpin RNA (shRNA) approach resulted in more severe liver disease [30]. These experiments demonstrate the fundamental protective role of autophagy in formation of ALD.

Collectively, these findings from hereditary and toxic liver diseases corroborate that autophagy as a cellular degradation and clearance pathway is critical for maintaining liver homeostasis, especially in conditions of hepatic insults.

## 4. Autophagy in Liver Metabolism and Fatty Liver Disease

The most common liver disease worldwide is non-alcoholic fatty liver disease (NAFLD), that is characterized by extrahepatic features of the metabolic syndrome (obesity, type 2 diabetes, dyslipidemia) and distinct hepatic histological features [31]. A fraction of these patients develop non-alcoholic steatohepatitis (NASH), characterized by steatosis, inflammation, and hepatocyte ballooning, and are at a particular risk for progressing towards fibrosis, cirrhosis, and HCC [32]. Autophagy is a central “recycling mechanism” in hepatocytes, evolutionarily evolved to provide energy and to salvage key metabolites for sustaining anabolism [33]. Autophagy is therefore a key mediator of liver metabolism and is dysregulated in NAFLD [10]. For instance, autophagy provides amino acids to cellular processes via protein degradation and recycling of cell organelles [33,34], mobilizes intracellular glycogen storages (“glycophagy”) in case of starvation [33], and breaks down lipid droplets (“lipophagy”), which increases intracellular triglyceride and free fatty acid concentrations [35]. High levels of energy substrates (e.g., ATP), insulin, or free fatty acids negatively regulate autophagy, while starvation is one of the strongest physiological activators of autophagy in hepatocytes [10]. Importantly, hepatic autophagy is decreased overall in association with conditions that predispose to NAFLD such as obesity and aging [36]. Although an extensive body of literature suggests that the pharmacological modulation of either autophagy directly or autophagy-related up- or downstream pathways could hold therapeutic potential in obesity, metabolic syndrome, or NAFLD/NASH [37], lifestyle interventions including fasting, dietary changes, and exercise may also be very potent inducers of beneficial autophagy-related changes in metabolism [38,39].

The multidomain adaptor protein p62/SQSTM1 is an important substrate for autophagy in hepatocytes, as it can interact with a large set of ligands, such as arginylated substrates [40]. More recent work indicates that p62/SQSTM1 is phosphorylated and accumulated upon lipotoxic stimuli, aggravating steatohepatitis and autophagy defects [41].

Due to the central role of autophagy for hepatocyte metabolism, relatively fewer data exist on the role of autophagy in non-parenchymal cells during NAFLD. However, autophagy is certainly one contributing factor in the inflammatory and pro-fibrogenic (see below) environment. For instance, fatty acids, particularly palmitic acid, are capable of activating hepatic macrophages via the transcription factor hypoxia-inducible factor 1 alpha (HIF-1α), leading to impaired autophagy and a more inflammatory macrophage phenotype (e.g., interleukin-1β) [42]. Thus, impaired autophagy may not only affect hepatocyte metabolism, but also aggravate inflammation in fatty liver disease.

## 5. Autophagy in Liver Fibrosis and Cirrhosis

**Figure 2:**
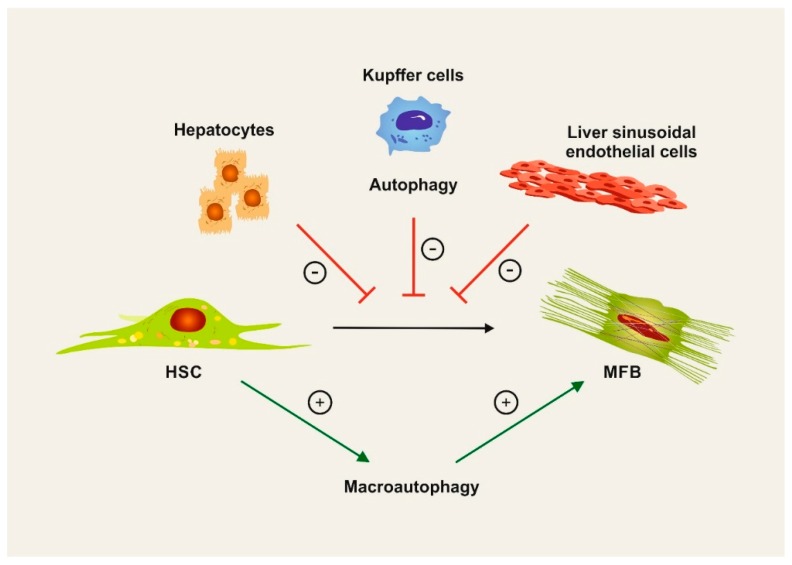
Cell type-specific functions of autophagy in liver fibrosis. Hepatic stellate cells (HSCs) transdifferentiate into collagen-producing myofibroblasts (MFB) in liver fibrosis. This process depends on macroautophagy, which provides energy for the HSC activation. On the contrary, autophagy maintains cellular homeostasis in hepatocytes, Kupffer cells (macrophages), and liver sinusoidal endothelial cells, thereby counteracting fibrogenesis in the liver.

The liver responds to chronic tissue injury by organ scarring, termed fibrosis, which may result in end-stage cirrhosis [43]. Liver fibrosis is characterized by concerted actions of non-parenchymal cells of the liver, particularly hepatic stellate cells, macrophages (including Kupffer cells), and endothelial cells [44]. Autophagy appears to be critically involved in the development of liver fibrosis, but has very different, opposing functions in specific cell types [45] (Figure 2).

The activation of hepatic stellate cells (HSCs) is central for liver fibrogenesis, because these cells transdifferentiate into myofibroblasts and represent the major extracellular matrix producing cells in the liver [46]. Activation of HSC depends on autophagy, because the autophagy-mediated degradation of lipid droplets stored in these cells provides energy supply and promotes fibrogenic cell functions [47]. Some of the molecular mechanisms have now been clarified. For instance, the micro-RNA miR-16 inhibits the expression of guanine nucleotide-binding α-subunit 12 (Gα_12_). During fibrogenesis, Gα_12_ is overexpressed and facilitates autophagy through ATG12-5 formation, thereby activating stellate cells [48]. Similar to hepatocytes, p62 is an autophagy substrate and thus negatively controls HSC activation [49]. Mechanistically, p62 promotes the formation of heterodimers between the vitamin D receptor (VDR) and retinoid X receptor-alpha (RXRα) that suppresses the fibrogenic response in HSC [49].

Autophagy-pathways in stellate cells can be induced via several signals. These include hypoxia-inducible factor-1alpha (Hif-1α) [50] and the potent fibrogenic cytokine transforming growth factor β1 (TGF-β1) [51], as well as the danger-associated pattern molecule high-mobility group box-1 (HMGB-1) [52]. Importantly, stellate cells also induce autophagy-related and fibrogenic genes in response to endoplasmatic reticulum (ER) stress signals [53], suggesting that autophagy indeed represents a central pathway of fibrogenic HSC activation. Consequently, the HSC-specific deletion of *Atg7* in mice attenuated liver fibrosis in chronic injury models [54]. Inhibiting autophagy by bafilomycin A1 decreased the proliferation and activation of primary mouse HSC in vitro, suggesting that autophagy inhibition in HSC could be an interesting therapeutic strategy [47].

While autophagy is profibrogenic in HSCs, autophagy seems to exert the opposite (i.e., antifibrotic) function in hepatic macrophages (Figure 2), the key cellular component of innate immune responses in the liver, during hepatofibrogenesis [55]. In mouse models of fibrosis, the macrophage-specific deletion of *Atg5* attenuated fibrogenesis [56]. Mechanistically, autophagy prevented the release of inflammatory cytokines, particularly interleukin-1, from hepatic macrophages, which subsequently reduced HSC activation [56]. Similarly, suppression of *Atg5* by a siRNA-approach confirmed that autophagy-deficient liver macrophages promote liver inflammation and fibrosis by enhancing mitochondrial ROS/NF-κB/IL-1α/β pathways [57]. Autophagy in hepatic macrophages is counteracted by the enzyme monoacylglycerol lipase that metabolises 2-arachidonoylglycerol into arachidonic acid for inflammatory macrophage activation [58].

Autophagy is also important for liver sinusoidal endothelial cells, which are a highly specialized endothelial cells separating the hepatocytes and hepatic stellate cells from the sinusoidal blood. These endothelial cells maintain the vascular tone, keep the stellate cells in a quiescent state, and promote tolerance in homeostasis [59] (Figure 2). Studies on isolated primary liver endothelial cells from either control or *Atg7*-deficient mice emphasized that autophagy is important for maintaining endothelial homeostasis [14]. In mouse and rat models of fibrosis induction, the selective loss of endothelial autophagy aggravated fibrosis by reduction in intrahepatic nitric oxide (NO) and impairment in handling oxidative stress, suggesting that autophagy is important for endothelial cell functions during chronic liver injury [14].

## 6. Autophagy in Liver Cancer

**Figure 3:**
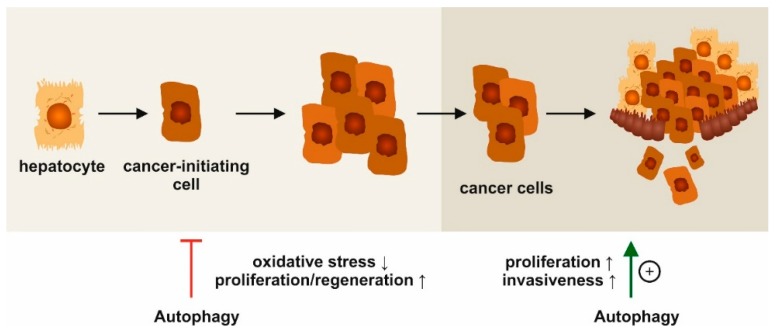
Stage-dependent functions of autophagy in hepatocellular carcinoma (HCC). Experimental data indicate opposing, stage-dependent functions of autophagy in HCC. At early stages, autophagy activation may reduce genotoxic stress and prevent tumor formation. At advanced stages with established tumors, autophagy is related to malignant proliferation and metastatic invasion.

Autophagy is important for hepatocyte homeostasis, as protein aggregates, lipid droplets, or organelles are eliminated via this pathway [60]. The lack of autophagy is associated with the development of spontaneous liver tumors (Figure 3), as demonstrated in liver- or hepatocyte-specific *Atg5*- and *Atg7*-knock-out mice [8]. These tumors indeed originate from autophagy-deficient hepatocytes and are characterized by aberrant p62 protein aggregation and mitochondrial swelling as well as increased genomic damage and oxidative stress responses [8]. On a molecular level, the elimination of p62 is a well-recognized anti-tumor function of autophagy [61], particularly in HCC [62]. In hepatoma cells, p62 accumulates, resulting in the persistent activation of nuclear factor (erythroid-derived 2)-like 2 (Nrf2) [63], which drives tumorigenesis in the liver in vivo [64]. Functionally, p62 not only activates Nrf2, but also mTORC1 and c-Myc, collectively promoting the survival of HCC-initiating cells [40].

Similarly, the oncogenic cell cycle regulator cyclin D1 is degraded by autophagy; defects in autophagy-dependent cyclin D1 degradation have been found in patients with HCC and confirmed in experimental HCC models in mice [65]. Autophagy also degrades the micro-RNA 224 (miR-224), which is linked to HCC development and poor prognosis in patients with hepatitis B virus (HBV) infections [66]. Moreover, autophagy-deficient hepatocytes release HMGB-1, which drives a proliferative ductular reaction as well as promotes tumorigenesis via the receptor for advanced glycation end product (RAGE) [67]. The exact molecular pathways of autophagy for HCC biology are the subject of many ongoing studies, which are summarized elsewhere [60,68,69].

There are controversial reports on the effects of drugs used in HCC regarding autophagy. An early study reported that sorafenib, a tyrosine-kinase inhibitor approved for the treatment of HCC, induced autophagy in HCC cell-lines [70]. However, autophagy has also been linked to sorafenib resistance [71,72]. Accordingly, the expression of autophagic markers in samples from HCC patients strongly correlate with annexin A3, which confers resistance to sorafenib as well as regorafenib [73]. Importantly, while autophagy apparently suppresses hepatocarcinogenesis, it is a pro-survival factor for cells and can therefore be also linked to tumor progression (Figure 3). This became evident from mouse models of metastatic liver cancer, in which autophagy favored disease progression [74,75]. Tumor cells may gain energy through autophagy, which favors their survival and migratory properties. Moreover, autophagy is associated with changes in the expression of cell adhesion molecules, which may facilitate the migration and invasiveness of malignantly transformed hepatocytes [69].

In addition to specific effects on hepatocarcinogenesis, autophagy in hepatocytes is also important for tumor surveillance in the whole body. However, while autophagy in hepatocytes mainly suppresses tumor formation in the liver [64], hepatocytic autophagy in general supports tumor growth [76]. This became evident in mice with a liver-specific deletion of either *Atg5* or *Atg7* that demonstrated an impaired growth of multiple allografted tumors. This observation was linked to the release of arginosuccinate synthase 1 from the liver and the subsequent degradation of circulating arginine, which is essential for tumor growth [9].

While most studies focused on the roles of autophagy in parenchymal cells for liver cancer, relatively little is known about the contribution of autophagy in non-parenchymal cells for HCC. During the preneoplastic state, autophagy in liver macrophages was found to suppress experimental hepatocarcinogenesis, mainly due to the anti-inflammatory role of autophagy in macrophages [57].

## 7. Therapeutic Implications and Outlook

Autophagy is a highly conserved process for degradation or recycling of cellular components and mobilization of energy substrates. Many drugs target directly or indirectly such processes, including the autophagy inducers carbamazepine, rapamycin, resveratrol, metformin, amitryptiline, or citalopram as well as inhibitors like choloroquine or hydroxycholoroquine. Many other more specific compounds are currently under development for various disease areas [77]. As described in our review, autophagy has both positive and negative roles in liver diseases, making it attractive but challenging to manipulate autophagy as a therapeutic approach in liver diseases. In this regard, two very exciting areas of research regarding autophagy-modulating therapies are metabolic [37] and malignant diseases [78]. Enhancing autophagy as a physiological process of reducing hepatocytic lipid accumulation and cellular stress signals emerges as an attractive target in NAFLD and NASH [10]. This could potentially include the repurposing of “known drugs” with an excellent safety profile. For instance, the autophagy activators carbamazepine and rapamycin decreased steatosis, dyslipidemia and insulin resistance in NAFLD mouse models [79]. However, enhancing autophagy should, ideally, target specifically parenchymal cells in the liver, muscle, and adipose tissue, to avoid the activation of fibrogenic HSC [54].

For liver fibrosis, many pharmacological approaches are currently being evaluated [80], but none of these approaches directly targets autophagy, likely due to the complex and cell type-specific role of autophagy during liver fibrosis. Based on the solid body of experimental data, the augmentation of autophagy in liver sinusoidal endothelial cells [14] as well as in macrophages [56] should be beneficial for fibrogenesis, particularly in early stages of the disease. On the other hand, autophagy is a key mechanism for the activation of hepatic stellate cells [46]. Thus, the HSC-specific inhibition of autophagy may be a potent antifibrotic strategy [47].

For HCC, it is intriguing to speculate that pharmacological induction of autophagy could limit tumor development. There are indications from mouse models that the pharmacological inducers amiodarone and rapamycin can prevent experimental hepatocarcinogenesis [66]. However, given the concomitant tumor-promoting functions of hepatocytic autophagy, it might be more advisable to target downstream effects, such as inhibiting phosphorylated p62-dependent Nrf2 activation [62]. In patients with metastatic HCC, it might be even advisable to inhibit autophagy, as this would likely increase the susceptibility to chemotherapy [45].

## 8. Conclusions

The deep mechanistic understanding of autophagy in the liver has uncovered a complex network of related molecular processes and the central role of autophagy for homeostasis and response to threats in the liver. Given the broad range of potential pharmacological and non-pharmacological (e.g., nutritional) interventions to target autophagy, it is intriguing to speculate on how to translate these findings into new therapeutics. Not surprisingly, autophagy is involved in disease-promoting as well as disease-limiting functions in a broad range of hepatological disorders. Cell-type- or disease-stage dependent effects can explain large parts of the dual functionality of autophagy. Thus, any autophagy-modulating intervention needs to be tailored to target the essential parenchymal or non-parenchymal cell type in the liver at the right moment of disease pathogenesis. With this caveat in mind, manifold options targeting autophagy for the treatment of hereditary, metabolic, toxic, fibrotic, or malignant liver disease may be anticipated in the future.
